# Conceptualization and content validation of the MEDication literacy assessment of geriatric patients and informal caregivers (MED-fLAG)

**DOI:** 10.1186/s41687-022-00495-2

**Published:** 2022-08-19

**Authors:** Jenny Gentizon, Mapi Fleury, Eric Pilet, Christophe Büla, Cedric Mabire

**Affiliations:** 1 Institute of Higher Education and Research in Healthcare, Lausanne University Hospital and University of Lausanne, Lausanne, Switzerland; 2 Service of Geriatric Medicine and Geriatric Rehabilitation, Lausanne University Hospital, Lausanne, Switzerland; 3 Oncology Department, Lausanne University Hospital, Lausanne, Switzerland; 4 Citizen’s College of Health Co-Researchers, attached to the ColLaboratoire, a Participatory and Collaboratory Action-Research Unit of Lausanne University, Lausanne, Switzerland

**Keywords:** Medication literacy, Assessment, Content validity, Older adults, Polypharmacy, Medication safety, Hospital discharge

## Abstract

**Background:**

The assessment of patients’ medication literacy skills (i.e., abilities to access, comprehend and interact with medication-related information) is an important step in assisting clinicians to plan for appropriate care. Despite several attempts by researchers to develop measures of medication literacy, an instrument tailored to the specific needs of older adults remains a significant shortfall. Therefore, an interprofessional team that included a citizen co-researcher conceptualized a new standardised measure of medication literacy—the MEDedication Literacy Assessment of Geriatric patients and informal caregivers (MED-fLAG). MED-fLAG was designed as a three-dimensional self-reported measure of functional, interactive and critical skills. This study describes the conceptualization process and provides the results of an evaluation of MED-fLAG’s content validity, acceptability, and feasibility during a hospital stay.

**Methods:**

MED-fLAG was developed in accordance with the guidance on scale development and standards for good content validity, by using the following steps: (I) conceptualization of a provisional version of MED-fLAG; (II) iterative qualitative evaluation of its content validity by older adults, informal caregivers and healthcare professionals.

**Results:**

The qualitative assessment of the initial 54-item MED-fLAG was conducted in 36 participants, namely 13 home-dwelling older adults and/or informal caregivers and 23 healthcare professionals. Six rounds of revisions were performed to achieve content validity and to propose a 56-item revised MED-fLAG. Participants reported benefits of using a standardized assessment of medication literacy during a hospital stay but warned about certain limitations and prerequisites. The extent to which MED-fLAG could be integrated into discharge planning needs to be further investigated.

**Conclusions:**

MED-fLAG is the first medication literacy measure tailored to the specific needs of older patients and informal caregivers. A unique feature of this measure is that it includes prescribed and non-prescribed medications, irrespective of the galenic form. Additional studies are required to evaluate the other measurement properties of MED-fLAG, and to reduce the number of items before considering its clinical application.

**Supplementary Information:**

The online version contains supplementary material available at 10.1186/s41687-022-00495-2.

## Plain English summary

On the basis of what has been written about medication literacy and the experiences of experts, we developed a new questionnaire to measure medication literacy (MED-fLAG) in older adults and/or informal caregivers. MED-fLAG was then submitted to older adults, informal caregivers and healthcare professionals to retrieve their feedback concerning the relevance, comprehensibility and exhaustiveness of the proposed items. In future, MED-fLAG will allow health professionals to evaluate medication literacy skills in older patients during hospitalization and/or in their informal caregivers when they are responsible for preparing or administering the medications, and then propose individualised support.

**Supplementary Information:**

The online version contains supplementary material available at 10.1186/s41687-022-00495-2.

## Background

The pattern of illness in older adults (≥ 65 years old) implies a higher prevalence of chronic conditions and a corresponding increase in medications. Older adults often deal with numerous long-term medications, potentially involving prescribed, non-prescribed and herbal products, with different dosages, galenic forms, and schedules of administration [1–5]. In such circumstances, self-management of medication can represent a complex self-care activity [6], requiring a high level of cognitive and social skills, that can be grouped under the concept of medication literacy skills [7].

Medication literacy was recently defined in a concept development study, as the degree to which older adults and/or informal caregivers can develop and maintain functional, interactive and critical skills [8]. These skills involve, for instance, the abilities to understand, prepare and self-administer medication (functional domain), to actively interact with healthcare providers, to express concerns and take part in decisions (interactive domain), and to seek reliable medication-related information, exert control over medication management and act appropriately in case of problems (critical domain).

Assessing and supporting sufficient medication literacy skills is a priority area in medication safety in high-risk situations, polypharmacy and transitions of care [9, 10]. Formal assessment of medication literacy during a hospital stay is a first necessary step to inform clinicians about the extent to which the medication regimen is adapted to the older patient’s skills, and assist them in optimizing this regimen and planning for individualized support [11–14]. Since medication management is the most common task reported by informal caregivers [15, 16], initiatives to prepare them for medication management and enhance their medication literacy skills appear to be of utmost importance.

Although several attempts have been made to develop standardised measures of medication literacy, these measures have so far been unsatisfactory [17, 18]. Their psychometric properties were found to be inconsistent, the rationale for skills considered essential for medication literacy assessment was poor, and none of these measures were developed for the specific needs of older adults [17, 18]. The lack of a medication literacy assessment specifically tailored to older adults is a significant shortfall that remains to be addressed, including its use among informal caregivers when they are responsible for medication preparation and administration.

Prior to their use in clinical practice, newly developed measures should demonstrate an adequate reflection of the concept, also referred to as content validity. Content validity is the first and most important psychometric property when developing new measures, as poor content validity would influence other aspects of validity, reliability and interpretability [19]. In addition, acceptability and feasibility aspects, often overlooked components of new patient-reported outcome measures [20], provide important information concerning the most suitable format and method of administering measures to support the delivery of care, as well as the potential response biases [21–23].

The objectives of this study were therefore to conceptualize and evaluate the content validity of a new medication literacy measure, the MEDication Literacy Assessment of Geriatric patients and informal caregivers (the MED-fLAG), including the preliminary acceptability and feasibility of its use during a hospital stay.

## Methods

The research methods were designed in accordance with guidance on scale development [24, 25] and the standards on content validity established by COSMIN—*COnsensus-based Standards for selection of health Measurement INstruments* [19]. This study included two steps: (I) conceptualization of a provisional version of MED-fLAG; (II) evaluation of its content validity, including the preliminary acceptability and feasibility of its use.

### Step I: conceptualization of MED-fLAG

In this first step, multiple information sources were used to apprehend the domains and subdomains underlying a measure of medication literacy in older adults. Three key domains of medication literacy – functional, interactive and critical – were identified in a previous concept development study, including a literature review and focus groups with hospital nurses [8]. During workshops, a multidisciplinary research team, including nurses, a geriatrician, a pharmacist and a citizen co-researcher (i.e., patient partner) were invited to reflect on what they would consider essential skills in the functional, interactive and critical domains, contributing to the clarification of subdomains. Then, the multidisciplinary research team developed an extensive item bank that aimed to cover the domains and subdomains of medication literacy. The citizen co-researcher, with comorbidities and a complex medication regimen, contributed to a sense check of the proposed items and to the breadth and depth of this conceptualisation phase [20]. The overall findings were used to shape the first version of MED-fLAG. At this stage, previously developed medication literacy measures [18] were considered in order to support item exhaustiveness.

The formulation of items and response options followed recommendations of general principles for writing items [21, 24, 26]. Based on literature about measurement instrument testing in older patient population, we avoided recall behaviours and used limited number of response options with a unidirectional scale [23, 27]. Items were worded for completion by older patients or their informal caregivers.

### Step II: content validity

#### Design

A qualitative method of data collection, namely cognitive interviewing, was chosen to achieve satisfactory content validity by asking older adults, informal caregivers and health professionals about the relevance, comprehensibility and exhaustiveness of the candidate items of MED-fLAG [19, 21]. Congruent with standards in psychometrics, content validity should target adequate content coverage by including many more items than it is expected to be found in the finalized measure [19, 24, 26]. Focus group approach was chosen to promote interaction and self-disclosure among participants. When focus group participation was not feasible for participants, alternative data collection methods were proposed, such as individual interviews or written evaluation (i.e., notes about problematic items on a paper-based version of the preliminary MED-fLAG).

#### Participants and setting

A convenience sample of home-dwelling older adults, informal caregivers and healthcare professionals was recruited in the French-speaking part of Switzerland between June and October 2021. Home-dwelling older adults and informal caregivers, not necessarily dyads, were recruited from patient and citizen associations through advertisements, and healthcare professionals were recruited from hospital and universities by advertisements and word of mouth. All participants had to be fluent in French. Home-dwelling older adults had to be ≥ 65 years old, and managing their medication for at least three months. Those who required assistance from healthcare professionals to manage their medication were excluded. Informal caregivers had to be ≥ 18 years old and responsible for preparing or administering medications on behalf of an older relative aged 65 years or more for at least three months. Prior experience with medication self-management of at least three months was used to gain insight from key informants about functional, interactive and critical medication literacy skills, as well as to increase information power [28]. Finally, any healthcare professionals (e.g., nurses, pharmacists, physicians) with experience in information or education of hospitalized older patients for self-medication management were invited to participate, independent of their professional background and position in their institution.

#### Data collection

Prior to the focus group sessions, participants were asked to perform a preparatory work by reading each item and taking notes about problematic items on a paper-based version of MED-fLAG. The instructions given were the following: *“With a red marker,(i) highlight words/phrases that are difficult to understand and should be rephrased, (ii) words/phrases that are unclear, ambiguous, imprecise”, “With a blue marker, (iii) highlight statements in the MED-fLAG that you assess as irrelevant, inadequate and should be removed, (iv) statements in the MED-fLAG that do not apply to your experience in managing medicines”, “In the "Comments" column, you can (v) propose additional statements”.*

Home-dwelling older adults and informal caregivers attended three focus group sessions and provided feedback for all items in the functional, interactive and critical medication literacy domains. As healthcare professionals were not available to participate in more than one focus group or interview, they were invited to provide general feedback on MED-fLAG and then focused on items integrated into a single domain, according to their primary field of involvement in assisting patients with the medication process. Thus, hospital nurses provided feedback on the functional medication literacy items, physicians discussed the interactive-related items and pharmacists revised the critical medication items.

The focus group and interview sessions were moderated by the first author. The cognitive interviewing technique was used to identify items with problematic comprehensibility and to gather participants’ experience in functional, interactive and critical domains, contributing to the relevance and exhaustiveness of the items [29, 30]. Moving from general to specific questions, participants were asked to think aloud and verbalize their thoughts. Examples of open-ended questions were as follows: *“What did you think when you first read this item?”, “Can you tell me in your own words what you understand by reading this item?”, “To what extent do you think that certain items are not relevant, not important?”, “In the light of your experience in medication management, to what extent should any items be added?”.*

In addition, the preliminary acceptability and feasibility of MED-fLAG during a hospital stay were explored. Derived from the literature, acceptability was defined as the extent to which MED-fLAG can be useful for the clinical decision-making process and would be ethically acceptable. Feasibility was defined as the extent to which the format of MED-fLAG and the way it is presented to end-users (paper or online) are suitable, as well as the more practical aspects that support its implementation in the hospital setting [20, 31]. Examples of open-ended questions were as follows: *"To what extent do you think some items could be offensive or that people could be uncomfortable answering them?", "To what extent could personal and/or hospitalization-related factors influence how people respond to the MED-fLAG?", "In your opinion, when is the best time to submit the MED-fLAG to patients and/or informal caregivers?", "Which format and mode of administration would you prefer (paper and pencil, electronic format on a tablet, face-to-face in an interview with a healthcare professional)?", "What suggestions do you have for facilitating future use of the MED-fLAG prior to hospital discharge?".*

At the end of data collection through focus groups and interviews with older adults, informal caregivers, and healthcare professionals, the revised MED-fLAG was presented to all participants through an online survey to collect their vote. Participants were asked to read each item and vote according to three options: (1) accepted as is; (2) rewording needed; (3) questionable relevance. They could also provide comments and narrative evaluation.

#### Data analysis

Qualitative data from participants were analysed and summarized in a standardized item tracking matrix by the first author (JG). Quotes from participants were used to support rewording of items when appropriate. A coding scheme was used for the categorization of types of problems reported on each item [29]: *Ambiguous, equivocal interpretation* was coded for items with ambiguous meaning or lacking precision; *Problems with wording* was coded items for difficult to understand (e.g., jargon); *Doubtful relevance, appropriateness* was coded for items with questionable importance or covering a different conceptual perspective than medication literacy; *Additional items* was coded for items that were proposed by participants. In addition, participants’ feedback was retrieved concerning the preliminary acceptability and feasibility of the MED-fLAG during a hospital stay. Thematic analysis was used to code the narratives of participants. Revisions of items were made according to the analysis.

A numerical endpoint for a satisfactory content validity was derived from the COSMIN guideline [19]. The items of MED-fLAG had to be rated as relevant and comprehensible by at least 85% of the participants. This was calculated from the online survey (i.e., items for which participants voted “Accepted as is”). In addition, exhaustiveness of each domain had to be sufficient in the proposed final version (i.e., no more than one or two items added, based on free text comments in the online survey).

### Ethical considerations

This project was approved by the Ethics Committee (ID 2021-0086). In accordance with the ethics committee’s requirements during the COVID-19 pandemic, focus groups and interviews were proposed to be conducted virtually. Consent to participate was obtained prior to focus groups/interviews. Each session was recorded. Home-dwelling older adults and informal caregivers received a gift card for their participation.

## Results

In the following results, we report the conceptualization of a provisional version of MED-fLAG (Step I) and evaluation of its content validity, including aspects of acceptability and feasibility (Step II).

### Step I: conceptualization of a provisional version of MED-fLAG

**Fig. 1:**
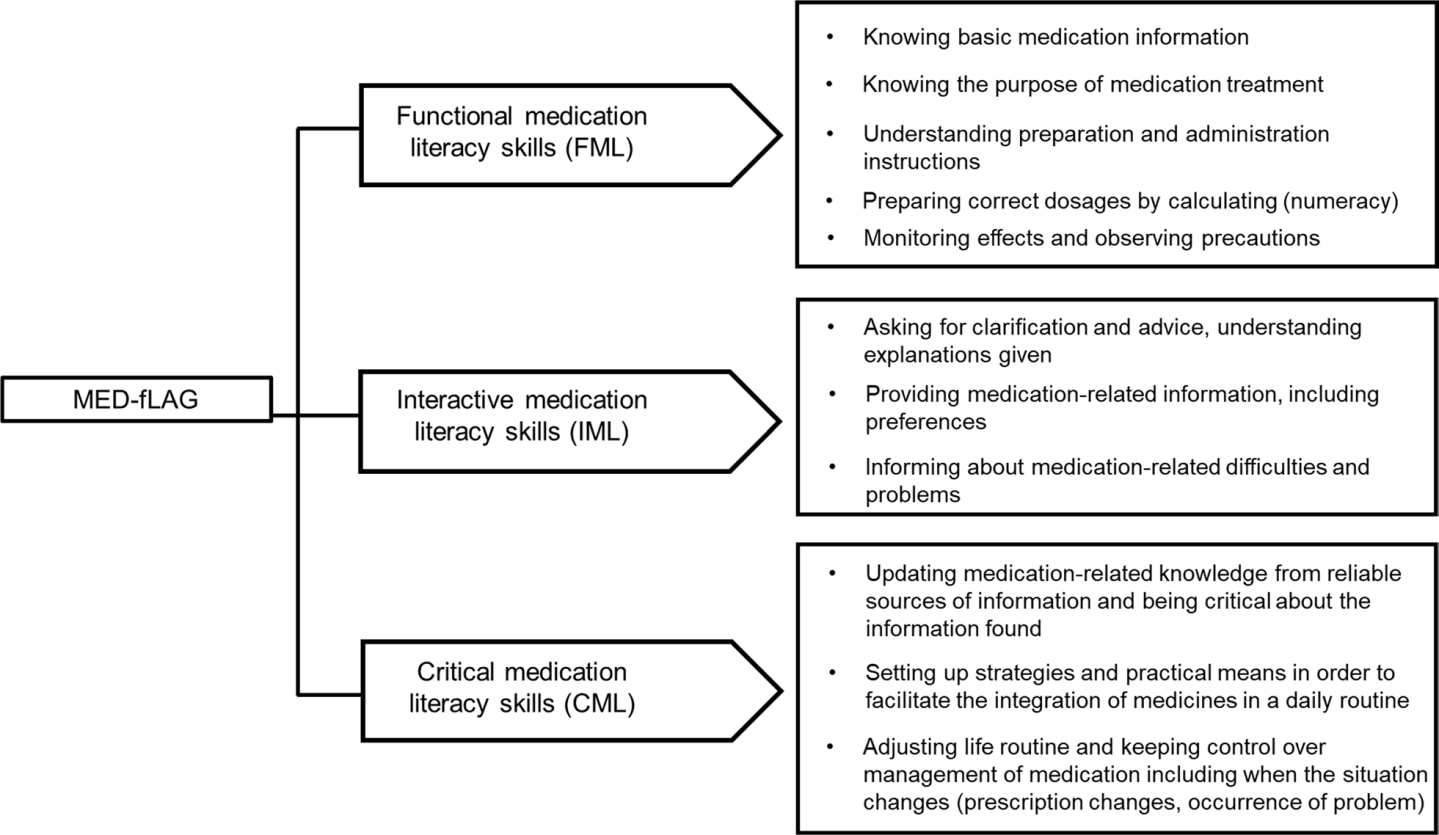
The medication literacy domains of functional (FML), interactive (IML) and critical skills (CML), and associated subdomains

Three domains (functional medication literacy [FML], interactive medication literacy [IML], critical medication literacy [CML]) and 11 subdomains were identified as conceptually relevant to cover medication literacy skills in hospitalized older patients and/or informal caregivers (Fig. 1).

A first draft of the measure was elaborated by compiling 54 items, among which 27 items covered FML, 17 covered IML, and 10 covered CML. Scoring options were graded as follows: (a) level of difficulty (Likert scale from 4 = not difficult at all to 1 = very difficult/impossible) and (b) frequency of actions (Likert scale from 4 = always to 1 = never). Higher MED-fLAG scores indicate higher medication literacy skills.

### Step II: content validity, acceptability and feasibility

A total of 36 participants were enrolled. In the older adult and/or caregiver group (N = 13; 36.1%), two participants (≥ 65 years of age) were responsible for their own medications and also for an older relative's medications, and one participant had responsibility for medications only for an older family member. The healthcare professional group (N = 23; 63.9%) included nurses (N = 5; 13.9%), pharmacists (N = 6; 16.7%) and physicians (N = 12; 33.3%). Four participants, namely two older adults, one pharmacist and one physician, could not attend the focus group session and were interviewed individually. A total of 10 focus groups and interviews were conducted. Characteristics of participants are described in Tables 1 and 2.

**Table 1:** Characteristics of home-dwelling older adults and/or informal caregivers (*N* = 13)

	*N* (%)	MEDIAN MIN–MAX
*Medication management responsibility*		
For self	10 (76.9)	
For self and an older relative ^a^	2 (15.4)	
Only for an older relative ^a^	1 (7.7)	
Age, years		71
		53–86
Female	5 (38.5)	
*Marital status*		
Single	1 (7.7)	
Married or partnered	2 (15.4)	
Divorced	10 (76.9)	
*Educational status*		
Secondary school	1 (7.7)	
Apprenticeship	8 (61.5)	
High school	1 (7.7)	
University	3 (23.1)	
*Number of medications*^*b*^		
< 5	4 (30.8)	
5–10	7 (53.8)	
> 10	2 (15.4)	
*Last hospitalization *^*c*^		
< 3 months	4 (26.7)	
3–6 months	1 (6.6)	
> 6 months	6 (40)	
Do not know, never	4 (26.7)	
*Last medication change *^*c*^		
< 3 months	4 (26.7)	
3–6 months	3 (20)	
> 6 months	6 (40)	
Do not know, never	2 (13.3)	

^a^Two participants (≥ 65 years of age) were responsible for their own medications and also for an older relative's medications, and one participant had responsibility for medications only of an older family member

^b^For the participants who were both responsible for the medication management of an older relative and for themselves, the total number of medications was summed

^c^A total of 15 observations are included, reporting information for respondents aged ≥ 65 years responsible for the medication management for themselves and for an older relative, and the information of one respondent who had a substitute role

**Table 2:** Characteristics of healthcare professionals (*N* = 23)

	*N* (%)	MEDIAN MIN–MAX
Female	17 (73.9)	
Professional experience, years		10
		1–28
*Setting*		
Hospital	20 (87)	
Other (community pharmacy, ambulatory care, university)	3 (13)	
*Field of practice*		
Internal medicine	1 (4.3)	
Surgery	2 (8.8)	
Neurology	1 (4.3)	
Dermatology	1 (4.3)	
Palliative care	1 (4.3)	
Geriatrics (including geriatric rehabilitation)	13 (56.6)	
Psychiatry	1 (4.3)	
Oncology	1 (4.3)	
Other	2 (8.8)	

**Fig. 2:**
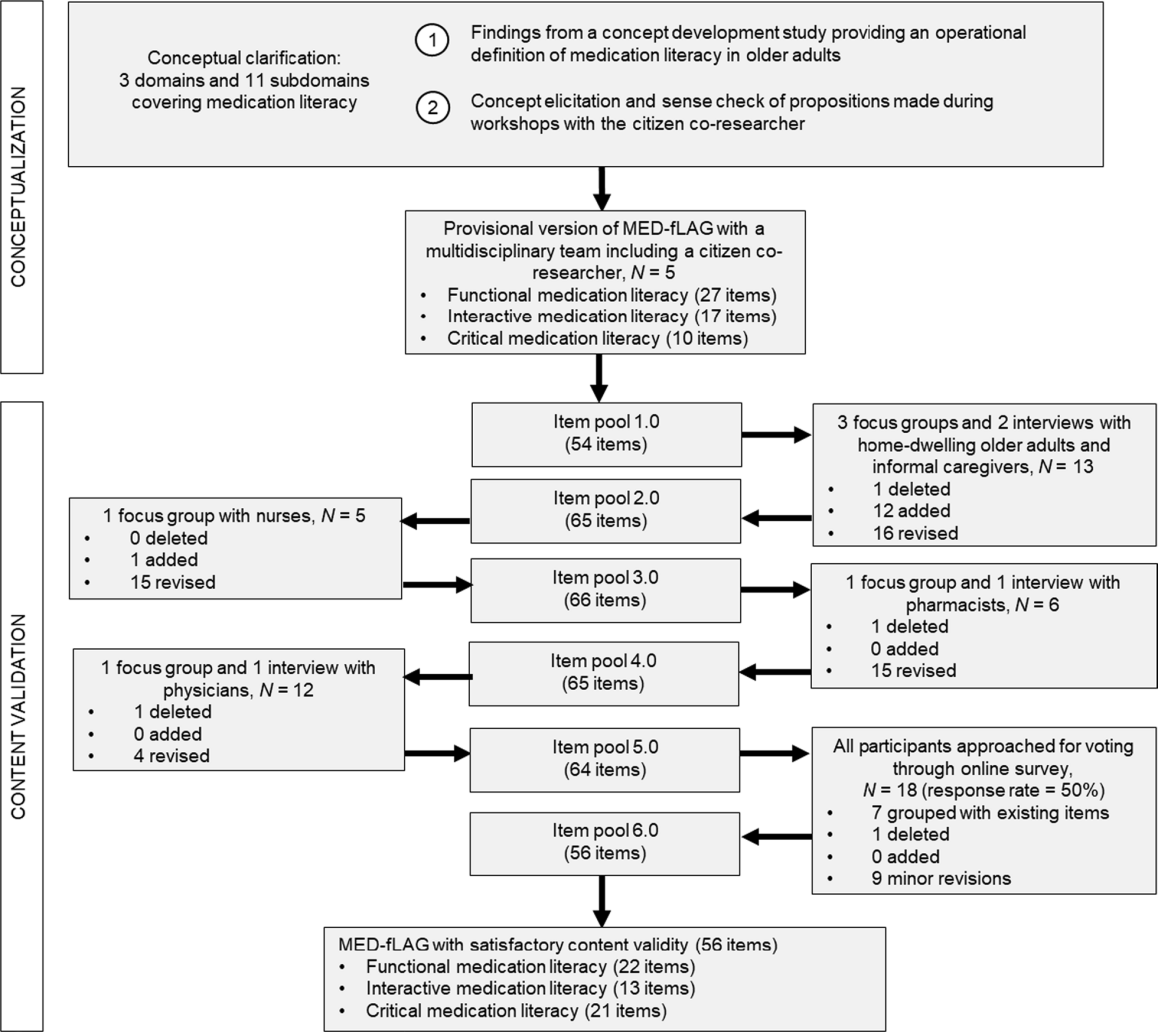
Data collection process to establish the content validity of MED-fLAG

The data collection process is illustrated in Fig. 2. A total of six rounds of revisions of the item pool were performed. After the fifth round of revisions, MED-fLAG was presented to all participants through an online survey. A total of 18 participants completed the evaluation of the final version (response rate of 50%). Participants were nine healthcare professionals (nurses N = 3, pharmacists N = 4, physicians N = 2), and nine older adults among whom two also had an informal caregiver role (medication management responsibility for self as an older adult N = 7, medication management responsibility for self as an older adult but also for an older family member as an informal caregiver N = 2).

From the online survey, fifty-nine items (92.2%) reached the satisfactory content validity endpoint (i.e., items “Accepted as is”), and no additional items were added. Based on free texts comments from participants, nine minor revisions were performed (i.e., rewording) and seven items were grouped with existing items (i.e., redundancy, similar conceptual perspective) and one was deleted because of its “Questionable relevance” (i.e., the item “…say where unused or expired medicines should be returned” appeared to evaluate environmental/recycling awareness more than knowledge and skills related to medication literacy).

At the end of the revision process, there was sufficient evidence of satisfactory content validity of the MED-fLAG, which included 56 items: 22 items in the functional domain, 13 in the interactive domain and 21 in the critical domain. (Examples of items included in MED-fLAG after content validation, see Additional file 1). With the aim of providing empirical evidence supporting the content validation process of MED-fLAG, we describe a selection of the item revisions in Table 3. Some participants’ quotes are used to support interpretations.

**Table 3:** Examples of item revisions made based on participants’ feedback on comprehensibility, relevance and exhaustiveness

N°	Original item	Type of problem	Illustrative quotes	Notes	Changes made (revised, deleted, added items)
*Item comprehensibility*					
1	[FML7] …Provide the names of the various prescribers who prescribed the medicines you manage	Problems with wording	“Sometimes it [MED-fLAG] is made up of big words and I don’t know what to make of it, I don’t understand that (prescribers and interaction) [Home-dwelling older adult 11]	While patients mostly refer to “doctors”, healthcare professionals were proposed to be more inclusive concerning other professionals authorized by law to prescribe medications. The item was made more precise	[FML7] …Give the names of the different doctors or other health professionals who prescribed the medicines you manage
2	[FML20] …Say whether any of the medicines you manage may cause problems of interaction with other medicines [FML21]…Say whether any of the medicines you manage may interact with food and drink	Problems with wording	“The medications to avoid (…) prohibited food or drinks” [Home-dwelling older adult 6]	The term "interaction" was identified by participants as a linguistic expression attributed to medical jargon. Items were reworded	[FML20] …Say whether certain medicines are incompatible with those you are managing [FML21] …Say whether certain foods or drinks should be avoided or are prohibited with the medicines you are managing (alcohol, grapefruit, juice, lactose-based food etc.)
3	[FML18] …Describe the negative effects that could occur, even when medicines are taken correctly	Ambiguous/equivocal interpretation	"Difficult to answer (…) I didn't feel any positive or negative effect (example about a medicine). I think it's difficult to describe the positive effects" [Home-dwelling older adult 1] "A medicine that is prescribed by a doctor must be discussed, what it is used for and the side effects" [Informal caregiver 1]	The term “negative effects” was found to be unclear. The term “side effects” appeared to be potentially more appropriate. The item was reworded	[FML18] …List the main side effects of medicines, i.e., effects that are not intended but may occur (headaches, nausea, diarrhoea, dizziness, etc.)
4	[IML23] …Ask questions about medicines [IML24] …Ask questions about changes to the current list of medicines	(Other: redundancy)	“I don't see the difference [between the items]” [Physician 2]	Conceptual overlap of statements in a few items. Items were clustered together and refined by using examples derived from participants’ dialogue	[IML23] …Ask healthcare professionals for additional information about the medicines you are managing (precautions, risks and benefits, changes to the current list of medicines, etc.)
*Item relevance*					
5	[CML54] …Identify lifestyle habits (physical activity, diet) that could have a beneficial influence on the health situation and the list of current medications	Doubtful relevance/appropriateness	“Seems to concern healthy lifestyle not medication” [Physician 1]	Seems to approach a different concept. The item was deleted	(Suppression of item)
6	[IML36] …Express your level of satisfaction with the information you received about medicines	Doubtful relevance/appropriateness	“This item concerns satisfaction with care rather than skills towards medication management” [Physician 1]	Seems to approach a different concept. The item was deleted	(Suppression of item)
7	[FML1] …Name all the medicines you manage, both prescription and non-prescription medication	Doubtful relevance/appropriateness	“Well at the moment…I have got seven or eight medicines to take every day and now I’ve got to take this one [new medicine]…I can't remember these names, I wrote it all down” [Home-dwelling older adult 5]	Some items imposed too high level of skill requirements for older patients and informal caregivers. Worded as is, this item implicitly means that the names of medication should be known by heart. This was found to be hardly achievable for polymedicated people (≥ 5 medications). Thus, participants thought that referring to a list was appropriate	[FML1] …Name both prescription and non-prescription medicines you are managing (either by heart or by using a list)
8	[FML18] …Describe the negative effects that could occur, even when medicines are taken correctly	Doubtful relevance/appropriateness	“I think that the hardest thing for any patient is trying to recognize it [side effect]…the doctor can’t expect to know what it is, unless he tells you… I’ve never had anyone coming back to me and explain the side effects” [Home-dwelling older adult and informal caregiver 4]	When they occur, side effects of medication could be difficult to discriminate from the symptoms related to the underlying disease. Nevertheless, home-dwelling older adults and informal caregivers mentioned that they wished to have more information concerning the “major” and “more important” side effects of their medication. Precision about “the main side effect” was added	[FML18] …List the main side effects of medicines, i.e., effects that are not intended but may occur (headaches, nausea, diarrhoea, dizziness, etc.)
9	[CML2] …Describe medicines by their appearance (colour and shape)	Doubtful relevance/appropriateness	“When I prepare my pillbox for the week, I take a look before closing each box and it's important for me to know the shape [of the medications]. Let's say one goes off [lost], it's interesting to know it by colour and shape it has” [Home-dwelling older adult 11]	For home-dwelling older adults, having an idea of medication appearance is a manner of checking medications before taking them, or to identify a medication that was dropped on the floor. However, to healthcare professionals, taken alone, this skill would not be a sufficient to ensure safe medication use. As this item was evidenced to be congruent with home-dwelling older adults’ experience, it was maintained	(Maintained as is)
*Item exhaustiveness*					
10	(None)	Additional items	“I had to find solutions by myself (…) I have a medication plan on my smartphone” [Home-dwelling older adult 1] “I have a medication plan on me, it is with the driving licence (…) I also prepare my medication for the week (pillbox prepared without the help of a healthcare professional” [Home-dwelling older adult 12]	Home-dwelling older adults and informal caregivers shared their experience about practical means and strategies that they use to facilitate integration of medication taking into their daily life, such as lists, notes, reminders, electronic devices. Narratives were used to create new items	[CML41] …Keep a list of the medicines you are managing (in your wallet, on your phone) [CML42] …Use a treatment plan that describes when and how each medication should be taken [CML44] …Use a pillbox that you prepare yourself for several days (without the help of a health professional)
11	(None)	Additional items	“My wife had cancer. At the beginning I spent time looking for information [medication-related] and organized things but now only after the list of medicines has changed” [Informal caregiver 1]	Home-dwelling older adults and informal caregivers shed light on challenges occurring along the continuum of care, notably with respect to medication changes, and the way they handle them. Proactive behaviours, focusing on seeking help and adjustments of their strategies and routines, were translated into new items	[CML52] …Adapt your daily routines after the list of medicines has changed (e.g., after hospitalization) [CML51] …Organize the storage of medicines, know when to go to the pharmacy after a prescription change
12	(None)	Additional items	“It can be difficult to open medication blisters and packaging” [Pharmacist 4]	Since medication literacy is confined to cognitive and social skills (i.e., not physical capabilities), difficulties in opening medication blisters and packaging were not added to MED-fLAG. Nevertheless, the capacity for individuals to cope with medication-related problems, including practical ones, is an important aspect of critical medication literacy	[CML54] …Get help from your family or people around you if you have difficulties with medications [CML55] …Get help from health professionals if you have problems with medications
13	(None)	Ambiguous/equivocal interpretation	“Herbal remedies that I take are not on my list [self-made list]” [Home-dwelling older adult 5] “The importance of safe practice with complementary medicine, for example, St. John's wort treatment with oral anticoagulant” [Pharmacist 2]	The term “medication” frequently cited across items appeared to be understood by home-dwelling older adults and informal caregivers as “prescribed medications”. Nevertheless, healthcare professionals underlined the importance of safe practice in using natural products. Items were created in order to raise awareness that natural products are not without risk	[FML2] …Name the natural products you manage, such as herbal treatments, homeopathy, food supplements (by heart or list) [IML24] …Ask healthcare professionals for advice about natural products, e.g., herbal remedies, homeopathy and food supplements
14	[CML44] …use a pillbox that you prepare yourself for several days, without the help of a health professional [FML2] …list the names of the natural remedies you manage, e.g. herbal treatments, homeopathy, dietary supplements (by heart or with a support such as a prescription list)	(Other: unsatisfactory response options)	“May be relevant [item] but not applicable to all” [Physician 1]	An additional response option was added because certain activities or actions are less common, i.e., would not be experienced by all MED-fLAG respondents	Response option added: Not applicable to my situation

### Acceptability and feasibility of MED-fLAG use during the hospital stay

Home-dwelling older patients, informal caregivers and healthcare professionals could all foresee potential benefits of using a standardized assessment of medication literacy during a hospital stay but warned about certain limitations and prerequisites. Participants underlined the perceived usefulness of the MED-fLAG but also the risk of embarrassment of certain items (acceptability), along with more practical aspects of its use in clinical settings, such as the timing, format of delivery, as well as well as local conditions to foster its implementation (feasibility).

#### Usefulness

All participants welcomed the value of MED-fLAG. They mentioned that MED-fLAG is a useful tool to prompt patient-clinician discussions about medications before hospital discharge, which in turn could assist clinicians in identifying when additional support is needed: *“It gives us an idea from the patients themselves about the importance and prevalence of problems as they see them. So I think that that would provide some very useful information as to how we can improve* [our services]*”* [Healthcare professional 13].

Some participants pointed out that items were not established at the same ‘difficulty’ level. One item was found to be easier, requiring fewer skills (FML3): “…describe medicines by their appearance (colour and shape)”. Others would require increasingly demanding cognitive skills (CML40): “…question reliability of information about medicines you find in the media, advertisements, health magazines, social networks” and (IML29): “…provide information about the effects observed, that has been experienced and that you think could be associated with the medicines”. This suggests that MED-fLAG could capture individuals with different performance levels, namely from low to high medication literacy skills. One proposition made was to consider repeated medication literacy assessments along the continuum of care to provide more accurate and reliable information about patients’ experience with medication, rather than a single measure at hospital discharge: *“Once might not give a clear snapshot… Whereas if you did it (assessment) four times a year… It would definitely give you more of an overview”* [Healthcare professional 5].

#### Risk of embarrassment

None of the items were found to be offensive, but those that were intended to report medication errors and omissions that had happened in the last few weeks were found to be potentially embarrassing. These items were clustered together to mitigate potential embarrassment and to limit the risk of response bias. In addition, participants warned about information that has to be given to future end-users (patients/informal caregivers) before the completion of MED-fLAG, including the aim of the assessment and the way this information will be used. Hospital discharge is a critical period, and patients could be overloaded with information, in a hurry to go home or concerned about consequences in reporting difficulties with medication management: *“People could wonder what will happen then with this data”* [Healthcare professional 7] and *“I probably wouldn’t let my doctor know what I’m going through”* [Home-dwelling older adult 1].

#### Timing

Participants agreed that emergency room and hospital admission were not appropriate settings and time to complete MED-fLAG. The main reason older adults would decline to complete a questionnaire would be if they do not feel well enough: *“If something gives me a shock (diagnosis) or makes me feel very stressed, my brain doesn’t function very well*” [Home-dwelling older adult 3].

The older adults’ participants mentioned their willingness to complete MED-fLAG as inpatients at the time of their discharge, as well as outpatients including during a visit to their general practitioner. Nevertheless, participants perceived that identifying the best timing to use MED-fLAG would constitute a challenge, because of the large variations between services to plan and prepare patients and their family for hospital discharge.

#### Format of delivery

Participants considered that the format of delivery (e.g., paper–pencil, electronic format or interview-based) should be adapted to each respondent’s preferences and abilities. Labelled categories were found to be more acceptable than a numeric scaling or pictorial icons, such as smiley faces. Pictorial icons were considered as infantilizing by home-dwelling older adults. Overall, their general recommendation was to provide adequate support to the patients with limited proficiency (i.e., people who are not fluent in French): *“You have to present things according to the patient's level of understanding”* [Healthcare professional 1].

#### Readiness of clinical settings

For older adults and informal caregivers, use of MED-fLAG during the hospital stay would require an improvement in the discharge preparation process. Readiness of services and healthcare professionals should be considered, together with implementation of a standardized assessment of medication literacy skills: *“Sometimes they (professionals) don’t realize the biggest things that are affecting us”* [Home-dwelling older adult 2] and *“They see you for five minutes (…)…the time factor…you are worrying that perhaps you’re taking up too much time”* [Home-dwelling older adult and informal caregiver 4].

## Discussion

This study describes the conceptualization of a new measure of medication literacy, as well as the evaluation of its content validity by home-dwelling older adults, informal caregivers and healthcare professionals. Six rounds of revisions were performed to achieve content validity and propose a 56-item revised MED-fLAG covering functional, interactive and critical medication literacy domains. The work presented here contributes significantly to the field of medication literacy in different ways.

Results of the conceptualization phase further enhance our understanding of the domains at stake when attempting to address issues related to medication literacy. The MED-fLAG underlines that older adults need extensive skills in functional, interactive and critical domains. These domains brings a more detailed perspective with respect to the patient work process involved in medication management, previously described as complex, cognitive, and collaborative [6]. These findings highlight that the previously developed Drug Regimen Unassisted Grading Scale as well as the Medication Management Instrument for Deficiencies in the Elderly, which focus on functional skills [32], would not be sufficient to estimate the medication literacy skills in older patients.

The qualitative approach used in this study allowed to precise and operationalise the critical medication literacy, derived from critical health literacy of the Nutbeam’s model [33]. While critical health literacy was repeatedly found vague and poorly operationalized [18, 34], our qualitative approach including a systematic evaluation of relevance, comprehensibility and exhaustiveness of items led to the creation of new items in the critical medication literacy domain. In MED-fLAG, these notably include the use of practical strategies to organise medications, including when the situation changes. These practical strategies and routines, developed by patients, were previously described as pragmatic ways to manage workload and to exert control over the situation [35, 36]. The current study acknowledges the role of patients in enhancing safety in the medication management chain [37–39].

MED-fLAG conceptualization resulted in a more comprehensive perspective of challenges encountered by older adults, as it integrates items for prescribed and non-prescribed medications, irrespective of the galenic form, including herbal remedies and food supplements. Despite many older individuals take herbals, nutriments, poly-vitamins [1, 2, 4] these are often overlooked for their importance and risks [35]. Previous medication literacy measures were confined to conventional medicines, except for one measuring medication literacy in herbal products [18, 40].

An additional contribution of MED-fLAG is to allow the assessment of the medication literacy skills of informal caregivers when they are responsible for preparing and administering medication to their older family member. Identification of their difficulties could allow clinicians to plan appropriate support, whether informal caregivers have to take on a gradual role in medication management or a more sudden one in the case of critical illness of their older relative, such as after hospital discharge [36, 41, 42].

Finally, the evaluation of MED-fLAG content validity was designed to address limitations described in previously developed measures [18]. In particular, the qualitative approach conducted among home-dwelling older adults, informal caregivers and healthcare professionals allowed the identification of problems with items that are usually invisible to researchers when using a quantitative approach, such as calculation of the content validity index. Most of the item-related problems were minor issues that could be solved with relatively small changes to the wording or clarification of the phrasing. A clear strength of the current work was the involvement of a patient representative whose contribution proved essential to capture the complexity of medication literacy skills and enhanced the overall rigor of the conceptualization of this new measure, as previously proposed [20].

Nevertheless, content validity incorporates more subjectivity than for other measurement properties, based on numerical endpoints [43]. For content validity, there is no proposed criteria to use as an endpoint. In the present study, qualitative evidence of content validity was used in conjunction with a numeric endpoint derived from COSMIN guidelines [19]. Although the establishment of satisfactory content validity is considered fundamental, clearer methodological procedures, including reporting guidelines, are needed to improve its estimation and trustworthiness [44]. Optimizing content validity procedures could be achieved by applying mixed method designs, in which the qualitative and quantitative methods inform each other [45, 46]. An exploratory sequential mixed-method research [47], for example, would first use a qualitative approach in a sample of end-users and then be complemented by a Delphi technique in a larger sample to quantify a degree of agreement, calculating the percentage of agreement on the relevance, exhaustiveness and comprehensibility concerning the final set of items. While different indices exist to quantify the degree of agreement among experts [48, 49], having additional guidance for the selection and the interpretation of appropriate index in content validity studies would be helpful.

Prior to MED-fLAG use in clinical practice, further evaluation of its measurement properties (i.e., validity, reliability and responsiveness) must be performed, along with descriptive statistics for interpretability of the scores (i.e., floor and ceiling effects across domains). The evaluation of the hypothesized dimensionality, also referred to structural validity, will allow a reduction in the number of items [50]. Future psychometric studies of MED-fLAG should consider the use of Item Response Theory (IRT), as suggested by previous research on health literacy instruments [51]. IRT allows to consider items with different ‘difficulty’ levels; more ‘difficult’ items would mean that patients need higher medication literacy skills. IRT could therefore be used to calibrate patients’ performance and the establishment of cut-off scores allowing the categorisation of individuals with different levels of medication literacy (i.e., low/adequate) [52, 53]. MED-fLAG scores’ reliability is another psychometric property that should be investigated in the future [21]. Participants showed concerns about consequences in reporting difficulties with medication management could potentially influence the way they answer to MED-fLAG questions. In such circumstances, any change in the MED-fLAG scores would not necessarily be due to a change in the patients’ medication literacy skills, but could be attributed to random errors, namely external factors, natural variation in the context and individual differences [21]. These findings provide essential information in designing research procedures of upcoming psychometric studies. Strategies to reduce random error include repeating measurements in the same individuals, conducting studies in large samples or removing the source of errors that could influence measurements. Increasing the control of hospitalization-related response bias could be achieved by providing participants with detailed information concerning the aim of the assessment and the way that the scores will be used, favouring anonymous participation in MED-fLAG psychometric studies, or testing the reliability and measurement error away from hospitalization (i.e., home-dwelling older adults) [21, 52]. The extent to which MED-fLAG could be integrated into the discharge planning needs to be further investigated.

There are some limitations in this study. Qualitative data were collected from home-dwelling older adults, not currently hospitalized. Because of the data collection procedures accorded by the ethics committee during COVID-19, we were unable to include currently hospitalized individuals, and participation of older individuals was confined to those who had confidence in using virtual tools. The characteristics of the included sample may therefore partially reflect the target patient population of MED-fLAG, and the medication literacy skills that appeared important to this cohort may differ in hospitalized patients.

Although previous research showed that online interviews could be a valuable alternative to face-to-face interviews to collect data [54], we cannot exclude that using virtual tools could produce a bias towards participants who are more skilled and/or educated. Younger age and higher educational achievement were found to correlate to higher eHealth literacy [55, 56]. Future studies should therefore consider purposeful sampling to mitigate selection bias, and target greater variation in individuals’ characteristics by using selected qualities, such as ethnicity, language, socioeconomic status, computer experience, and severity of condition [57].

In addition, this study’s population was defined by the chronological age (≥ 65 years and older). However, the chronological age is insufficient to describe the medical, functional, emotional, and social changes that an individual may be experiencing. Adults age in different patterns and with different health trajectories, and older adults are in fact a heterogeneous population. To better reflect the wide range of geriatric patients that will potentially complete MED-fLAG in its future clinical application, upcoming psychometric studies should use purposeful sampling by targeting maximum variation in older individuals’ characteristics [57]. The use of aging stratifications that combine chronological age, functional status, disease burden and geriatric syndromes, could enhance representativeness of the heterogeneity of the older population [58].

Finally, despite several attempts to recruit informal caregivers, a small number of participants endorsing this role (N = 3) was included. A limited insight of specific medication literacy skills and issues encountered by informal caregivers could therefore remain, potentially influencing the generalizability of findings. Future studies should consider a more systematic application of *Patient and Informal Caregiver Participation In Research* [59] by involving a group of older adults and informal caregivers in the research team. Information power could be further enhanced by purposefully involving individuals with a variety of experience in medication self-management [28].

## Conclusions

Built on a qualitative approach that included home-dwelling older adults, informal caregivers and healthcare professionals, this study established a content-valid measure of medication literacy: the MEDication Literacy Assessment of Geriatric patients and informal caregivers (MED-fLAG). The period of hospitalisation could provide an opportunity to identify older individuals, or their informal caregiver, with insufficient medication literacy, and provide a red flag to propose an individualised support and eventually perform revisions of the medication list, contributing to the prevention of medication-related problems. The next step in the development of MED-fLAG is to investigate its other psychometric properties in a large sample of older individuals and informal caregivers, and reduce the number of its items before considering a clinical application.

## Supplementary Information

**Additional file 1. Appendix A.** Selection of items included in MED-fLAG, after its content validation (free translation from French).

## Data Availability

Examples of items included in MED- fLAG are available in Additional file 1: Appendix A.

## Abbreviations

MED-fLAG: MEDication Literacy Assessment of Geriatric patients and informal caregivers
COSMIN: COnsensus-based Standards for selection of health Measurement INstruments
