# Supplementary material for: Conceptualization and content validation of the MEDication literacy assessment of geriatric patients and informal caregivers (MED-fLAG)
Source: J Patient Rep Outcomes. 2022 Aug 19;6:87. doi: 10.1186/s41687-022-00495-2 (PMC9389474; doi:10.1186/s41687-022-00495-2)
Supplement: Supplementary file 1 — Additional file 1. Appendix A. Selection of items included in MED-fLAG, after its content validation (free translation from French). [file 41687_2022_495_MOESM1_ESM.docx]

**Appendix A.**

Selection of items included in MED-fLAG, after its content validation (free translation from French)

| **Functional medication literacy** [FML] is the degree to which patients or their informal caregivers: a) have basic knowledge about the medications; b) know the purpose; c) understand the instructions related to the preparation and administration ; d) are able prepare correct dosages (i.e., calculating); and e) are able to monitor of the effects and observe precautions. | | |
| --- | --- | --- |
|  |  |  |
| **Suppose you have to describe what you know about the medication you manage, and what you have understood about the instructions.   How difficult is it for you to:** (4 = Not difficult at all; 3 = Somewhat difficult; 2 = Difficult; 1 = Very difficult or impossible; NA = Not applicable to my situation) | | |
| **Knowing basic medication information (6)** | **FML1** | ...list the names of all the medicines you manage, both prescription and non-prescription (either by heart or with the help of a support such as a prescription list) |
|  | **FML3** | ...describe medicines by their appearance (colour and shape) |
| **Knowing the purpose of the medication treatment (4)** | **FML7** | ...explain in your own words why each medicine has been prescribed |
| **Understanding preparation and administration instructions (5)** | **FML11** | ...say which medicine should be taken before, during or after a meal |
|  | **FML14** | …say how long each medicine should be taken |
| **Preparing correct dosages by calculating (numeracy) (2)** | **FML16** | ...prepare the correct dosage of medicines, e.g., counting the number of tablets, the number of drops, or using a graduated syringe (or measuring cup) |
| **Monitoring effects and observing precautions (5)** | **FML18** | ...say the main side effects of medicines, i.e. effects that are not intended but that could occur (headaches, nausea, diarrhoea, dizziness, etc.) |
|  | **FML21** | ...say whether certain foods or drinks should be avoided or are prohibited with the medicines you manage (alcohol, grapefruit juice, lactose-based foods, etc.) |
| **Interactive medication literacy** [IML] is the degree to which individuals: a) ask for clarification and understand explanations given by healthcare professionals; b) provide medication-related information; and c) inform about medication-related difficulties and problems. | | |
| **Suppose you have to describe your ease of communication with health professionals about the medicines you manage  How difficult is it for you to:** (4 = Not difficult at all; 3 = Somewhat difficult; 2 = Difficult; 1 = Very difficult or impossible; NA = Not applicable to my situation) | | |
| **Asking for clarification and advice, understanding explanations given (3)** | **IML23** | ...ask health professionals for additional information about the medicines you manage (precautions to take, risks and benefits, changes to the current medicine list, etc.) |
|  | **IML24** | ...ask health professionals for advice about natural remedies, e.g., herbal remedies, homeopathy and food supplements |
|  | **IML25** | ...understand information about medicines given by health professionals |
| **Providing medication-related information, including preferences (5)** | **IML26** | ...describe the latest changes that have been made in the list of current medicines (new medicines initiated, those removed, changes in dosage) |
|  | **IML28** | ...express your interest in natural remedies such as herbal remedies, homeopathy, food supplements |
| **Informing about medication-related difficulties and problems (5)** | **IML31** | ... discuss any difficulties in following the medication plan, or in following the instructions |
|  | **IML33** | ...tell a health professional if you have stopped or changed the dosage of a medicine, or if you are thinking of doing so |
|  | **IML35** | ...seek professional advice for information about medicines that you have found in the media, advertisements, health magazines or social networks |
| **Critical medication literacy** [CML] is the degree to which individuals: a) update their medication-related knowledge from reliable sources of information; b) set up strategies and practical means to integrate medication taking in a daily routine; and c) adjust and keep the control over their medication including when the situation changes. | | |
| **Suppose you have to describe how you update your knowledge about the medicines you manage, and how you use reliable sources of information**  **Do you usually :** (4 = Always; 3 = Often; 2 = Sometimes; 1 = Never; NA = Not applicable to my situation) | | |
| **Updating medication-related knowledge from reliable sources of information and being critical about the information found (5)** | **CML36** | ...read the package insert given with the medicine box |
|  | **CML38** | ...seek advice from a health professional before taking any over-the-counter medicines, including natural herbal remedies, homeopathy and food supplements |
|  | **CML40** | ...question the reliability of information about medicines that you find in the media, advertisements, health magazines or social networks |
| **Suppose you have to describe your strategies, your daily routines that help you with the management of the medicines  Do you usually:** (4 = Always; 3 = Often; 2 = Sometimes; 1 = Never; NA = Not applicable to my situation) | | |
| **Setting up strategies and practical means in order to facilitate the integration of medicines in a daily routine (8)** | **CML41** | …carry a list of the medicines you manage (in your wallet, on your phone) |
|  | **CML42** | ...use a treatment plan that describes the medicines that need to be taken |
|  | **CML43** | ...use routines and strategies that allow you to check that the medicines have not been forgotten (alert on the phone, note each time the medicines are taken, ask a family member to check, etc.) |
|  | **CML48** | ...go to the same pharmacy to get medicines (having the medication file in the same unique pharmacy) |
| **Suppose you have to describe how you keep the control over the management of the medication, including when the prescription is changed or in case of problems with the medication**  **How difficult is it for you to:** (4 = Not difficult at all; 3 = Somewhat difficult; 2 = Difficult; 1 = Very difficult or impossible; NA = Not applicable to my situation) | | |
| **Adjusting life routine and keeping control over management of medication including when the situation changes (prescription changes, occurrence of problems) (8)** | **CML50** | ...know when to schedule a prescription renewal by the physician or other health professionals |
|  | **CML52** | ...adapt your daily routines after the list of medicines has changed (e.g., after hospitalisation) |
|  | **CML54** | ...get help from your family or people around you if you have difficulties with medicines you manage |
|  | **CML56** | ......know which healthcare professional to contact if you have problems with the medicines you manage |
